# Supplementary material for: Cellular and genetic mechanisms that shape the development and evolution of tail vertebral proportion in mice and jerboas
Source: Nat Commun. 2025 Oct 10;16:9014. doi: 10.1038/s41467-025-63606-9 (PMC12514186; doi:10.1038/s41467-025-63606-9)
Supplement: Supplementary file 1 — Supplementary Information [file 41467_2025_63606_MOESM1_ESM.pdf]

SUPPLEMENTARY FIGURES

(Supplementary tables can be found separately in Supplementary Data)

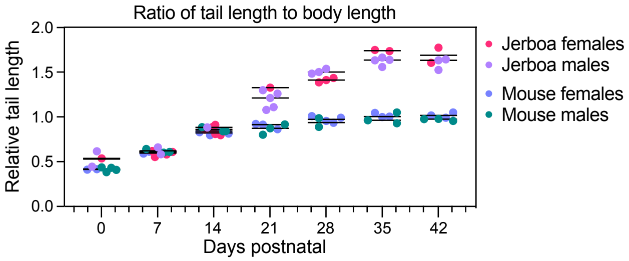

Supplemental Figure 1. Tail lengths do not vary between male and female mice through development. A two-way ANOVA was used to compare the effect of sex on relative tail length in mice and jerboas and no significant difference was found (ANOVA $p = 0.1649$, Shapiro-Wilk test of normality $p = 0.9907$). Six male and female animals were measured for each time point, except jerboa P0 and P42 where n=5.

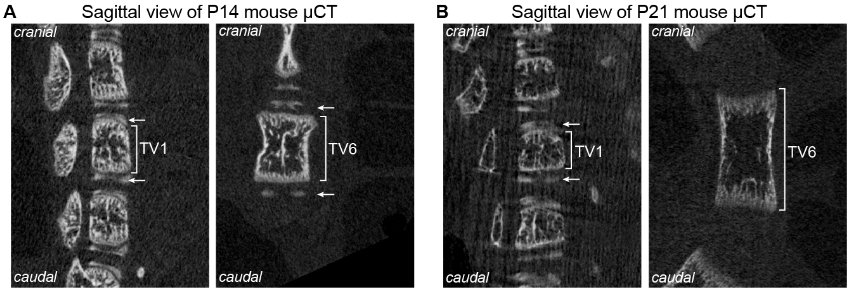

Supplemental Figure 2. Endplate formation is evident in µCT scans of both species, and endplate ossification is delayed in jerboa TV6. Vertebral epiphyses (endplates, marked with arrows) form at the cranial and caudal ends of the vertebral diaphysis (brackets) by day P14 in mouse and in jerboa TV1 but not yet TV6 at P21. Images are screen captures of µCT scans.

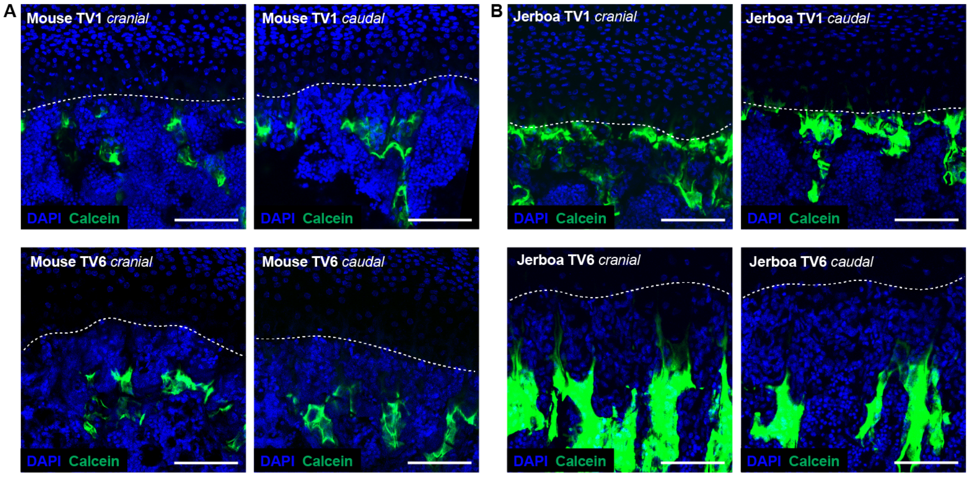

Supplemental Figure 3. Visualization of daily growth using calcein dynamic histomorphology. Representative maximum intensity projections of calcein (green) labeling against nuclei (DAPI; blue). The chondro-osseus junction is identified with a white dotted line. Identity of each growth cartilage is indicated in the top left of every frame. Scale bar is 100µm. Mouse TV1 caudal and mouse TV6 cranial and caudal n = 8; mouse TV1 cranial and all jerboa TV1 and TV6 cartilages n = 9; mixed male and female animals.

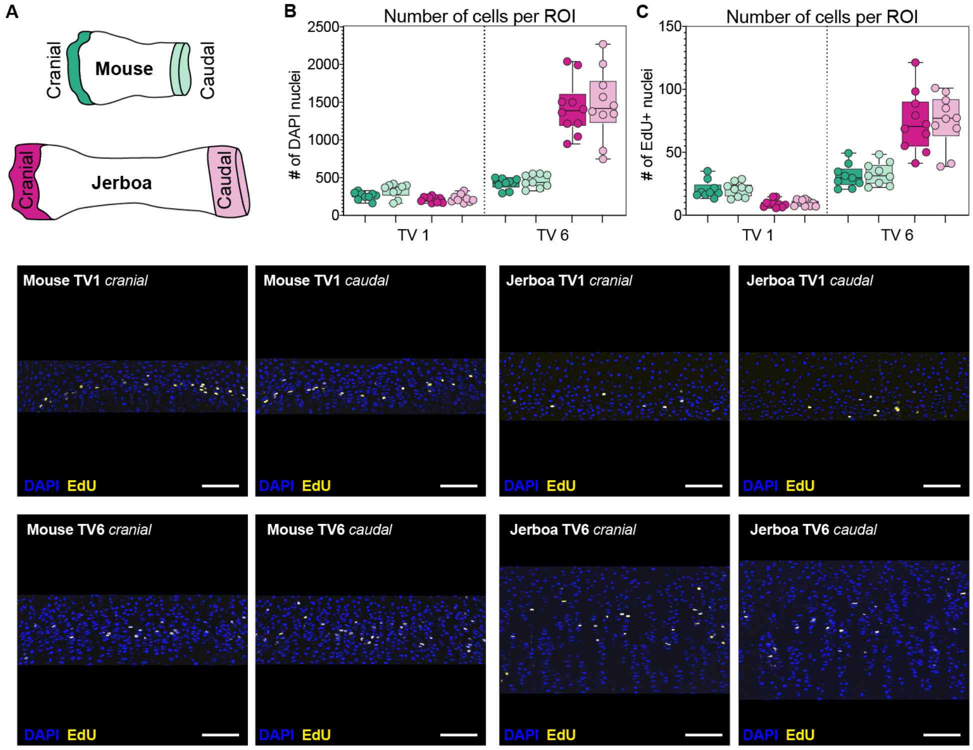

Supplemental Figure 4. Visualization of EdU positive cells to determine proliferation index. (A) Drawing of mouse and jerboa vertebrae with corresponding color scheme. (B) Number of DAPI+ cells per region of interest (ROI). (C) Number of EdU+ cells per ROI. (B-C) Each individual measured is indicated by a dot overlaying a box and whiskers plot (25th to 75th percentile box, min and max whiskers, median indicated in centre line). (D) Representative ROI including whole proliferation zone. EdU (yellow) labeling against nuclei (DAPI; blue). Identity of each growth cartilage is indicated in the top left of every frame. Scale bar is 100 µm. For mouse cartilages n = 9, and n = 8 for all jerboa cartilages.

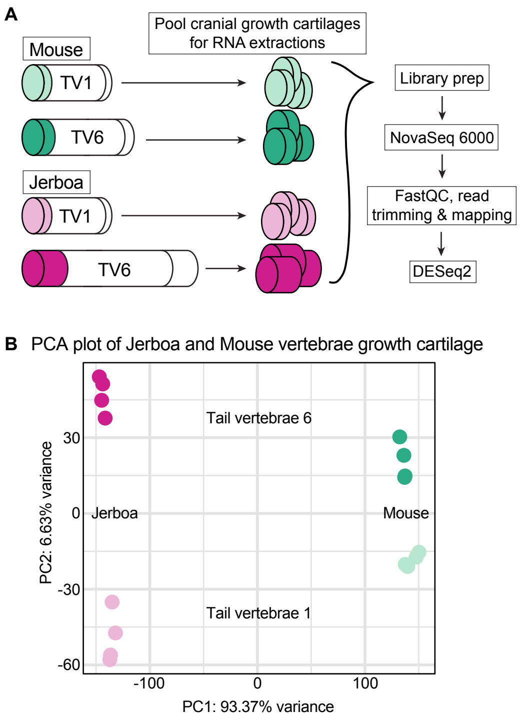

Supplemental Figure 5. Experimental design of intersectional interspecies transcriptomics. (A) Schematic outlining experimental design for the RNA-sequencing experiments. (B) Principal components analysis of mouse and jerboa TV1 and TV6 replicates (n = 4).

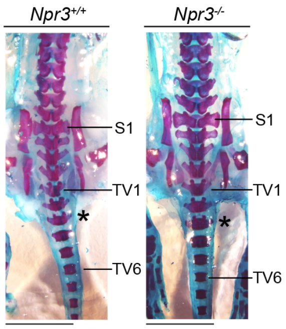

Supplemental Figure 6. Vertebral identity/shape is unchanged by loss of NPR3. Representative alizarin-stained axial skeletons of *Npr3*$^{-/-}$ and wildtype siblings at postnatal day 7. First sacral (S1) vertebra and TV1 and TV6 are indicated. Asterisk labels last vertebra with superior articular processes, which corresponds with the last vertebra with zygapophyseal articulation in the adult mouse tail. Scale bar is 5mm. n = 3 for both genotypes.
