# Supplementary material for: Cellular and genetic mechanisms that shape the development and evolution of tail vertebral proportion in mice and jerboas
Source: Nat Commun. 2025 Oct 10;16:9014. doi: 10.1038/s41467-025-63606-9 (PMC12514186; doi:10.1038/s41467-025-63606-9)
Supplement: Supplementary file 3 — Description of Additional Supplementary Files [file 41467_2025_63606_MOESM3_ESM.pdf]

Description of Additional Supplementary Files

File name: Supplementary Data 1

Description: 1864 genes are significantly differentially expressed between jerboa and mouse TV6 (padj<0.05) but not between species in TV1

File name: Supplementary Data 2

Description: All genes that are significantly differentially expressed between jerboa and mouse TV6 and between jerboa and mouse TV1

File name: Supplementary Data 3

Description: 421 disproportionately differentially expressed genes

File name: Supplementary Data 4

Description: Intra-species comparison within jerboa revealed that 7,911 genes are significantly differentially expressed between TV6 and TV1

File name: Supplementary Data 5

Description: 1,454 are differentially expressed in both datasets with the sign value consistently correlating with the most rapid rate of elongation in jerboa TV6

File name: Supplementary Data 6

Description: Summary of genes that have previously reported phenotypes related to body length or growth rates, or that have reported “short tail” or “long tail” mutant phenotypes in the Mouse Genome Informatics (MGI) database.

File name: Supplementary Data 7

Description: Disproportionately differentially expressed genes that correlate with jerboa TV6 elongation were intersected with 5 datasets
