# Supplementary material for: Cellular and genetic mechanisms that shape the development and evolution of tail vertebral proportion in mice and jerboas
Source: Nat Commun. 2025 Oct 10;16:9014. doi: 10.1038/s41467-025-63606-9 (PMC12514186; doi:10.1038/s41467-025-63606-9)
Supplement: Supplementary file 5 — Reporting Summary [file 41467_2025_63606_MOESM5_ESM.pdf]

nature portfolio

Corresponding author(s): Ceri J Weber and Kimberly L Cooper

Last updated by author(s): Nov 8, 2025

Reporting Summary

Nature Portfolio wishes to improve the reproducibility of the work that we publish. This form provides structure for consistency and transparency in reporting. For further information on Nature Portfolio policies, see our Editorial Policies and the Editorial Policy Checklist.

Statistics

For all statistical analyses, confirm that the following items are present in the figure legend, table legend, main text, or Methods section.

n/a	Confirmed	
☐	☒	The exact sample size (*n*) for each experimental group/condition, given as a discrete number and unit of measurement
☐	☒	A statement on whether measurements were taken from distinct samples or whether the same sample was measured repeatedly
☐	☒	The statistical test(s) used AND whether they are one- or two-sided<br>*Only common tests should be described solely by name; describe more complex techniques in the Methods section.*
☐	☒	A description of all covariates tested
☐	☒	A description of any assumptions or corrections, such as tests of normality and adjustment for multiple comparisons
☐	☒	A full description of the statistical parameters including central tendency (e.g. means) or other basic estimates (e.g. regression coefficient) AND variation (e.g. standard deviation) or associated estimates of uncertainty (e.g. confidence intervals)
☐	☒	For null hypothesis testing, the test statistic (e.g. *F*, *t*, *r*) with confidence intervals, effect sizes, degrees of freedom and *P* value noted<br>*Give P values as exact values whenever suitable.*
☒	☐	For Bayesian analysis, information on the choice of priors and Markov chain Monte Carlo settings
☒	☐	For hierarchical and complex designs, identification of the appropriate level for tests and full reporting of outcomes
☒	☐	Estimates of effect sizes (e.g. Cohen's *d*, Pearson's *r*), indicating how they were calculated

Our web collection on statistics for biologists contains articles on many of the points above.

Software and code

Policy information about availability of computer code

Data collection	FIJI, InteredgeDistance Macro for FIJI, DataViewer by Bruker, Python3, code written by co-author AJW (available on Dryad), FLUOVIEW software, cellSENS software
Data analysis	DEseq2 v1.44.0, STAR v2.7.10b, TOGA v1.1.6, Cutadapt, FastQC v0.12.1, TrimGalore v0.6.10, ggplot2 v3.5.2, dplyr v1.1.4, plotly v4.11.0, clusterProfiler v4.12.6, Hrbrthemes v0.8.7, paleteer v1.3.0, BiocManager v1.30.25, GeneOverlap v1.40.0, gprofiler v0.2.3, tidyr v1.3.1, RColorBrewer v1.1-3, viridis v0.6.5, reshape2 v1.4.4, Graphpad Prism software, Adobe Creative Cloud software (Illustrator and Photoshop)

For manuscripts utilizing custom algorithms or software that are central to the research but not yet described in published literature, software must be made available to editors and reviewers. We strongly encourage code deposition in a community repository (e.g. GitHub). See the Nature Portfolio guidelines for submitting code & software for further information.

Data

Policy information about availability of data

All manuscripts must include a data availability statement. This statement should provide the following information, where applicable:

- Accession codes, unique identifiers, or web links for publicly available datasets
- A description of any restrictions on data availability
- For clinical datasets or third party data, please ensure that the statement adheres to our policy

The RNA sequencing data generated in this study have been deposited in the GEO database under accession code GSE299515 [https://www.ncbi.nlm.nih.gov/gds/?term=GSE299515]. The processed RNA-seq data, 1:1 and 1:0 gtf annotation files generated with TOGA as well as gene lengths for these annotations are available on

Dryad [https://datadryad.org/dataset/doi:10.5061/dryad.70rxwdc92]. Raw images and histological measurements are also available on Dryad [https://datadryad.org/dataset/doi:10.5061/dryad.70rxwdc92]. Normalized measurements and gene subsets used to generate the graphs in Figures 1-5, S1, and S4 are provided in the Supplementary Data and Source Data files. Due to the size of the raw µCT files, these data are not yet available on a public repository. We have uploaded compressed files to a read only OneDrive and will grant access to data upon request.

Research involving human participants, their data, or biological material

Policy information about studies with human participants or human data. See also policy information about sex, gender (identity/presentation), and sexual orientation and race, ethnicity and racism.

Reporting on sex and gender	*Use the terms sex (biological attribute) and gender (shaped by social and cultural circumstances) carefully in order to avoid confusing both terms. Indicate if findings apply to only one sex or gender; describe whether sex and gender were considered in study design; whether sex and/or gender was determined based on self-reporting or assigned and methods used.*<br>*Provide in the source data disaggregated sex and gender data, where this information has been collected, and if consent has been obtained for sharing of individual-level data; provide overall numbers in this Reporting Summary. Please state if this information has not been collected.*<br>*Report sex- and gender-based analyses where performed, justify reasons for lack of sex- and gender-based analysis.*
Reporting on race, ethnicity, or other socially relevant groupings	*Please specify the socially constructed or socially relevant categorization variable(s) used in your manuscript and explain why they were used. Please note that such variables should not be used as proxies for other socially constructed/relevant variables (for example, race or ethnicity should not be used as a proxy for socioeconomic status).*<br>*Provide clear definitions of the relevant terms used, how they were provided (by the participants/respondents, the researchers, or third parties), and the method(s) used to classify people into the different categories (e.g. self-report, census or administrative data, social media data, etc.)*<br>*Please provide details about how you controlled for confounding variables in your analyses.*
Population characteristics	*Describe the covariate-relevant population characteristics of the human research participants (e.g. age, genotypic information, past and current diagnosis and treatment categories). If you filled out the behavioural & social sciences study design questions and have nothing to add here, write "See above."*
Recruitment	*Describe how participants were recruited. Outline any potential self-selection bias or other biases that may be present and how these are likely to impact results.*
Ethics oversight	*Identify the organization(s) that approved the study protocol.*

Note that full information on the approval of the study protocol must also be provided in the manuscript.

Field-specific reporting

Please select the one below that is the best fit for your research. If you are not sure, read the appropriate sections before making your selection.

☒ Life sciences ☐ Behavioural & social sciences ☐ Ecological, evolutionary & environmental sciences

For a reference copy of the document with all sections, see nature.com/documents/nr-reporting-summary-flat.pdf

Life sciences study design

All studies must disclose on these points even when the disclosure is negative.

Sample size	We used a sample size calculator to determine that we required at least six individuals, per group, to statistically power our histological comparisons. We are limited by the number of jerboa pups born; we collected up to nine pups for these experiments and therefore matched the mouse samples. We used a two-way ANOVA to test the effect of sex on tail elongation through postnatal growth and found no effect at any developmental stage. Therefore, we randomly assigned male/female animals to groups and have reported the sexes of all animals collected in the methods section.
Data exclusions	We discarded RNA-seq libraries that did not meet threshold QC scores. We did not analyze tissue sections that were torn or disrupted either during sectioning or staining.
Replication	Biological and technical replicates were performed for each experiment and are reported in the methods section. No replicates were omitted.
Randomization	Only healthy pups from mouse and jerboa litters were prepared for experiments. Male and female animals were assigned randomly as sex does not effect tail length.
Blinding	Histological and EdU images were blinded for tissue measurements with an assigned ID number applied to the image upon capture. The ID numbers were not replaced with animal information until data analysis was complete. uCT files were labeled with ID numbers after collection so genotype or age/sex was blinded during vertebral measurements.

Reporting for specific materials, systems and methods

We require information from authors about some types of materials, experimental systems and methods used in many studies. Here, indicate whether each material, system or method listed is relevant to your study. If you are not sure if a list item applies to your research, read the appropriate section before selecting a response.

Materials & experimental systems

n/a	Involved in the study
☐	☒ Antibodies
☒	☐ Eukaryotic cell lines
☒	☐ Palaeontology and archaeology
☐	☒ Animals and other organisms
☒	☐ Clinical data
☒	☐ Dual use research of concern
☒	☐ Plants

Methods

n/a	Involved in the study
☒	☐ ChIP-seq
☒	☐ Flow cytometry
☒	☐ MRI-based neuroimaging

Antibodies

Antibodies used	NPR3 anti-Rabbit polyclonal antibody, 1:500, Invitrogen, Cat# PA5-96947. Alexa Fluor 594 Goat Anti-Rabbit IgG (H&L) Cat# ab150080. Click-iTTM EdU Kit, Alexa Fluor 647 Cat# C10340.
Validation	The NPR3 antibody has been used for IHC in two recent manuscripts (DOI:10.1155/2020/8831735 and DOI:10.1155/2020/8831735). The supplier reports reactivity in human, mouse, and rat. We used the same concentration as reported in previous manuscripts.

Animals and other research organisms

Policy information about studies involving animals; ARRIVE guidelines recommended for reporting animal research, and Sex and Gender in Research

Laboratory animals	Mus musculus CD-1 (age postnatal day 0 to postnatal day 42) from Charles River Laboratories<br>Mus musculus C57BL/6 (breeding with Npr3-/-; aged <6 months) from Charles River Laboratories<br>Mus musculus NPR3 mutant mice MGI:2158355 (age postnatal day 7 and postnatal day 42) from Dr. Jeffery Olgin's laboratory at University of California San Francisco<br>Jac jaculus (age postnatal day 0 to postnatal day 42) - colony derived from animals imported from Egypt in 2008 by K.L.C.
Wild animals	*Provide details on animals observed in or captured in the field; report species and age where possible. Describe how animals were caught and transported and what happened to captive animals after the study (if killed, explain why and describe method; if released, say where and when) OR state that the study did not involve wild animals.*
Reporting on sex	We used a two-way ANOVA to test the effect of sex on tail elongation through postnatal growth of both mouse and jerboa and found no effect at any developmental stage. Therefore, we randomly assigned male/female animals to groups and have reported the sexes of all animals collected in the methods section.
Field-collected samples	*For laboratory work with field-collected samples, describe all relevant parameters such as housing, maintenance, temperature, photoperiod and end-of-experiment protocol OR state that the study did not involve samples collected from the field.*
Ethics oversight	*Identify the organization(s) that approved or provided guidance on the study protocol, OR state that no ethical approval or guidance was required and explain why not.*

Note that full information on the approval of the study protocol must also be provided in the manuscript.

Plants

Seed stocks	*Report on the source of all seed stocks or other plant material used. If applicable, state the seed stock centre and catalogue number. If plant specimens were collected from the field, describe the collection location, date and sampling procedures.*
Novel plant genotypes	*Describe the methods by which all novel plant genotypes were produced. This includes those generated by transgenic approaches, gene editing, chemical/radiation-based mutagenesis and hybridization. For transgenic lines, describe the transformation method, the number of independent lines analyzed and the generation upon which experiments were performed. For gene-edited lines, describe the editor used, the endogenous sequence targeted for editing, the targeting guide RNA sequence (if applicable) and how the editor was applied.*
Authentication	*Describe any authentication procedures for each seed stock used or novel genotype generated. Describe any experiments used to assess the effect of a mutation and, where applicable, how potential secondary effects (e.g. second site T-DNA insertions, mosiacism, off-target gene editing) were examined.*
